# Activating Partnership Assets to Produce Synergy in Primary Health Care: A Mixed Methods Study

**DOI:** 10.3390/healthcare9081060

**Published:** 2021-08-18

**Authors:** Ekaterina Loban, Catherine Scott, Virginia Lewis, Susan Law, Jeannie Haggerty

**Affiliations:** 1 St. Mary’s Research Centre, Montreal, QC H3T 1M5, Canada; jeannie.haggerty@mcgill.ca; 2 Department of Family Medicine, McGill University, Montreal, QC H3S 1Z1, Canada; 3 Department of Community Health Sciences, University of Calgary, Calgary, AB T2N 4Z6, Canada; cmscott@ucalgary.ca; 4 Australian Institute for Primary Care & Ageing, La Trobe University, Melbourne, VIC 3086, Australia; V.Lewis@latrobe.edu.au; 5 Institute of Health Policy, Management and Evaluation, University of Toronto, Toronto, ON M5T 3M6, Canada; susan.law@utoronto.ca

**Keywords:** partnerships, primary health care, partnership synergy, organizational transformation, health system improvement

## Abstract

Partnerships are an important mechanism to tackle complex problems that extend beyond traditional organizational divides. Partnerships are widely endorsed, but there is a need to strengthen the evidence base relating to claims of their effectiveness. This article presents findings from a mixed methods study conducted with the aim of understanding partnership processes and how various partnership factors contribute to partnership effectiveness. The study involved five multi-stakeholder partnerships in Canada and Australia working towards improving accessibility to primary health care for vulnerable populations. Qualitative data were collected through the observation of 14 partnership meetings and individual semi-structured interviews (*n* = 16) and informed the adaptation of an existing Partnership Self-Assessment Tool. The instrument was administered to five partnerships (*n* = 54). The results highlight partnership complexity and the dynamic and contingent nature of partnership processes. Synergistic action among multiple stakeholders was achieved through enabling processes at the interpersonal, operational and system levels. Synergy was associated with partnership leadership, administration and management, decision-making, the ability of partnerships to optimize the involvement of partners and the sufficiency of non-financial resources. The Partnership Synergy framework was useful in assessing the intermediate outcomes of ongoing partnerships when it was too early to assess the achievement of long-term intended outcomes.

## 1. Introduction

Despite the widespread endorsement of a partnership approach indicating that collaboration is generally a “good” thing in the context of complex problems, there is a need to strengthen the evidence base relating to claims of its effectiveness [1,2,3,4]. In practice, partnerships that bring different organizations and individuals together can generate a great deal of frustration, and those involved frequently struggle to achieve measurable, beneficial outcomes [5,6,7,8,9]. Estimates from formal evaluations suggest that up to 70% of alliances fail, and those that survive are frequently unable to reach their full potential [8,10,11]. These problems are not surprising given that partnerships are resource intensive, time consuming and require governance, procedures and processes that are very different from the ways independent organizations are run [8]. More research is therefore required to explore the factors, both positive and negative, that influence partnerships and mitigate success; otherwise, advocacy for partnerships will remain “a rhetorical issue with no basis in reality” [10].

This article presents findings from a mixed methods study of multi-stakeholder partnerships involving decision makers, academic representatives, clinicians, health system administrators, patient partners and representatives of health and social service organizations providing services to vulnerable populations with an interest in improving primary health care (PHC) accessibility. The study was conducted between 2016 and 2018 with the aim of understanding partnership processes and how various partnership factors contribute to partnership effectiveness within the context of addressing complex issues in PHC. We define a multi-stakeholder partnership as a complex human system based on voluntary collaborative relationships among stakeholders who agree to work together towards a common purpose, to combine competencies, resources and responsibilities and to share risks and benefits (adapted from [11]).

The focus of the study was on partnerships involving multiple stakeholders across organizations—transcending the boundaries between clinical practice, research, health system management and health care policy—collaborating towards a common goal of transforming PHC. In this study, we used a two-phase, exploratory sequential mixed methods design, embedded in a larger Canada–Australia research and evaluation project, to gain a comprehensive understanding of partnership processes, the interplay of partnership factors and the achievement of intended outcomes. This research adds depth to understanding the processes and approaches that support partnerships and assesses the relevance of the multi-stakeholder partnership approach in PHC. Equally, this research demonstrates how the application of theoretical frameworks can facilitate a deeper understanding of partnerships. 

### 1.1. Primary Health Care

PHC service delivery is the cornerstone of a high-performing health care system [12,13,14]. Robust PHC serves the following core functions: accessibility, continuity, comprehensiveness and coordination [15]. Among Organization for Economic Co-Operation and Development (OECD) countries, PHC is the first point of access to the health care system. It encompasses continuous and comprehensive care across diverse curative, preventative, educational and rehabilitation services with a person (micro), community (meso) and population (macro) orientation [13,16,17]. The centrality of PHC is underscored by its strategic coordination role that has a ripple effect on other parts of the health care system [18]. Advantages to having a comprehensive, responsive, high-quality PHC include improved population health outcomes, reduced inequities, improved patient and provider experience and satisfaction, lower health system costs and more robust health care systems overall [12,13].

Many countries have seen major improvements in the health of their populations over recent decades. However, progress has not been even across various dimensions relevant to PHC. Accessibility, in particular first-contact accessibility, is an ongoing concern in PHC [18]. Important disparities in equitable access to PHC remain, and there is evidence that gaps among social groups within countries have widened [14]. Several studies [19,20] have demonstrated that these gaps translate into unmet health care needs, delays in obtaining treatment, greater use of emergency departments and hospitalization and poorer health status for these patients. In addition, the resultant disparities present ethical challenges and place an unnecessary economic burden on publicly funded health systems. It is estimated that the direct economic burden of socioeconomic inequalities in health in Canada amounts to approximately $6.2 billion annually or over 14% of total expenditures on acute care inpatient hospitalizations, prescription medication and physician consultations [21]. In light of the realization of system deficiencies and rising health care expenditures, PHC in OECD countries has recently been the focus of a series of reform and reorganization efforts [22,23].

Canada and Australia have health care systems that aim to provide comprehensive, universal and accessible health care based on equal access for equal need. However, a review of the literature from both countries indicates that the goal of equitable access has not been uniformly achieved, particularly when it comes to lower income members of society such as Indigenous groups and refugees [24]. Both countries have undertaken initiatives to strengthen the PHC sector following findings of commissioned inquiries that highlighted suboptimal performance on many PHC access and quality indicators [25,26,27,28]. Many of these reform efforts have emphasized collaboration and partnerships among health care organizations, regional health authorities and other stakeholders as a way of achieving the goal of comprehensive PHC and delivering better care and implementation of policy [29,30]. This interest in partnerships has been reinforced by published evidence that partnerships involving multiple stakeholders can help people and organizations generate outcomes that are greater than those that can be achieved working independently [8,31,32]. 

One of the recent international initiatives to address the challenges related to equitable access to PHC was the Innovative Models Promoting Access-to-Care Transformation (IMPACT) program [33]. It was a 5-year (2013–2018) Canadian–Australian funded research program that saw the development of local partnerships as a strategy to enhance access to comprehensive PHC for vulnerable populations. Within the context of IMPACT, vulnerable populations were defined as community members whose demographic, geographic, economic, social and/or cultural characteristics compromised their access to PHC and limited their capacity to maintain health and advocate for themselves in the context of a complex health care system [33]. Six local partnerships in three Australian states (i.e., New South Wales, Victoria and South Australia) and three Canadian provinces (i.e., Ontario, Quebec and Alberta) used a common approach to implement six different interventions designed to address a priority gap in access to appropriate PHC. The interventions ranged considerably in focus and mechanisms (see Table 1). The partnerships involved decision makers, researchers, health and social services providers and administrators as well as members of vulnerable communities facing complex challenges to the delivery of community-based PHC. The partnerships identified priority access needs of vulnerable communities, informed the design of interventions based on the local context, engaged in deliberative processes for collective decision-making, and evaluated both the interventions and the partnerships themselves. The work reported here was conducted within the framework of the IMPACT program.

### 1.2. Partnership Evaluation and Partnership Synergy

Literature from diverse fields describes what constitutes effective as opposed to ineffective partnerships, and outlines strategies to enhance collaborative processes and increase partnership effectiveness [9,36,37,38,39,40]. This literature offers a variety of definitions and conceptualizations of partnership effectiveness. The prevailing approach is to assess partnership effectiveness in terms of its ability to achieve planned outcomes [41], also referred to as “results” or “consequences” [42]. These intended outcomes could be short-term, such as increased knowledge of stakeholders, intermediate, such as capacity building and buy-in, and long-term such as changes in community programs, policies and practices and improvements in health indicators [42,43]. 

An alternative approach to assessing partnership effectiveness is to focus on effective partnership as an end in itself, and as an intermediate outcome leading to the specific goals of the partnership. The assumption here is that partnerships that have a high level of internal functioning will be more likely to achieve their intended long-term outcomes [36]. The functioning of ongoing partnerships was the focus of this study, and we differentiated between the effectiveness of processes and outcomes. We captured information about the quality of the processes and relationships between partners and the health of the partnership on the one hand, and specific outcomes that could be attributed to the work of the partnerships. In addition, we collected information on the benefits of participating in partnerships for the stakeholders’ respective organizations, in line with the literature that identifies outcomes to partners as an important third strand of effectiveness, different from the strategic goals of the partnership [44]. 

To assess how a partnership is performing and to identify strengths and areas for improvement, strategies for measuring these multiple partnership dimensions and evaluating progress towards intended outcomes are needed. Partnership evaluation literature offers partnership measurement tools, notably self-assessment tools, that help organizations assess how the partnership is evolving and to stay accountable to the partnership’s stakeholders and funding bodies [45,46,47]. However, there is still ambiguity regarding the relationship among the various factors of a partnership’s work and effectiveness, particularly which partnership factors are more critical to partnership effectiveness [8,48]. Given the complexity and dynamic nature of partnerships and intractable problems they seek to address, inferring causal relationships between different partnership factors and effectiveness and identifying what percentage of the outcome is attributable to which factor is challenging [36,37]. This challenge is common in evaluating complex human systems that entail a web of interacting feedback loops where cause and effect are not close in time and space [49,50].

The Partnership Synergy framework aims to explain the link between partnership factors and partnership effectiveness [8]. Synergistic partnerships are high-performing partnerships that have created added value by leveraging the resources and capabilities of their partners. We define partnership synergy as the combined effect of complementary tangible and intangible partnership assets and enabling processes that gives partnerships unique advantages over the work of individual people or organizations working towards the same goals (adapted from [8]). Partnership intangible or “soft” assets include human, informational and organizational resources, such as knowledge, competencies, connections, culture, data and information, whereas tangible assets include material and financial resources such as space and funding [51]. While it is widely acknowledged in the field of management that tangible assets are the critical building blocks of organizations and companies, the rapid expansion of the knowledge economy has shed more light on the importance of “intangibles” and the benefits that they generate [51,52,53]. In fact, it has recently been argued that it is the intangible assets, such as skilled employees and unique know-how, that give companies their competitive edge [52]. In the field of partnerships, the critical role of both tangible and intangible assets has been highlighted in prior research [8,54]. It has also been suggested that intangible assets may constitute the outputs of partnership work, as health partnerships entail knowledge-generating activities and do not produce goods but rather knowledge, understanding and relationships [53]. For the purposes of this investigation, we considered intangible assets, produced by partnerships, such as learning, only insofar as they are reinvested back into the partnership and enhance partnership work and the achievement of intended outcomes. 

Certain partnership processes enable partnerships to leverage resources successfully and mobilize the complementary knowledge and expertise of all the partners to achieve partnership synergy [6,50]. Examples of enabling processes include leadership, administration and decision-making processes as well as the degree to which a partnership optimizes the involvement of its partners [8,50]. The literature identifying these key precipitators of partnership synergy is growing [6,50,55,56]. While it is common to refer to these precipitators more broadly as “partnership functioning factors”, we make a distinction between partnership assets that we view as partnership inputs and partnership enabling processes that act upon inputs to produce intended outcomes (adapted from [57]). 

It has been proposed to measure partnership assets (such as the sufficiency of resources) and enabling processes, along with partnership synergy, as a predictor of partnership effectiveness [8]. Two validated scales measure partnership synergy: the Weiss et al. scale [50] and the Jones synergy scale [58]. These scales measure partnership synergy in different ways: the Weiss et al. scale, which looks at synergy as a product of a partnership that has achieved its full potential, and the Jones synergy scale that measures synergy as both a partnership process or experience and a partnership product [58]. Both synergy scales are embedded in instruments that include other measures of different partnership factors. The Weiss et al. synergy scale is embedded in the Partnership Self-Assessment Tool (PSAT) [59], and both Weiss et al.’s and Jones’ synergy scales are incorporated in the Partner Questionnaire [6].

We adopted the Partnership Synergy framework to look at the formation and processes of IMPACT program’s multi-stakeholder partnerships. We selected the PSAT with some nuances from the Partner Questionnaire. 

## 2. Materials and Methods

### 2.1. Study Design

**Figure 1:**
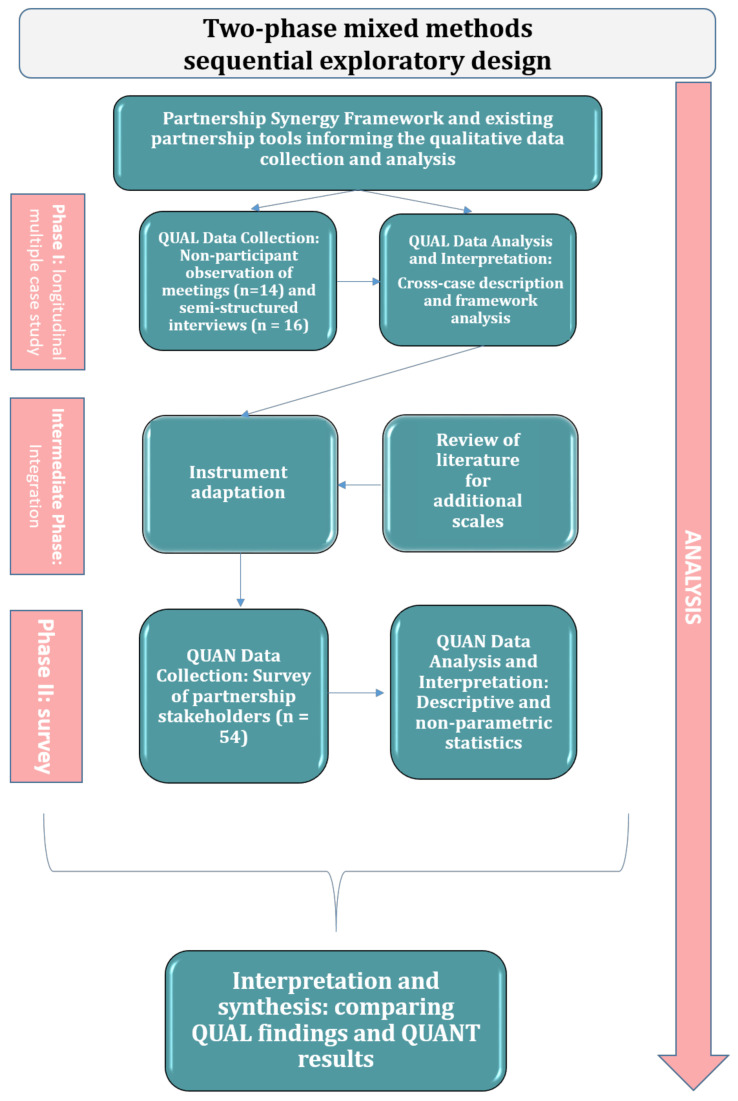
Mixed methods sequential exploratory design diagram.

The study entailed a two-phase mixed methods sequential exploratory design [60]. A qualitative longitudinal case study [61] (*n* = 2) was followed by a cross-sectional survey of IMPACT collaborative local partnerships (*n* = 5). Figure 1 illustrates the mixed methods design. The qualitative phase entailed exploring, in a smaller sample, the different manifestations of partnership synergy, the types of partnership assets and broad categories of partnership processes relevant to multi-stakeholder partnerships PHC [62]. Based on the qualitative findings, the quantitative phase included measuring, in a larger sample of partnerships, the achievement of partnership synergy and exploring associations between partnership synergy, partnership assets and partnership processes [63]. Qualitative findings and quantitative results were subsequently integrated to obtain a deeper understanding of the multi-stakeholder partnership phenomenon [60]. 

### 2.2. Background Work

Preliminary data gathering for the project involved the review of minutes of meetings, protocols and reports produced within the scope of the IMPACT program and, more specifically, the two local partnerships, Primary Care Connection and Community Health Resources. The review of documents provided data on the operational elements, participants’ roles and responsibilities, the objectives and intended outcomes of each initiative, and how the objectives and the involvement of different stakeholders evolved since the start of the IMPACT research program in 2013. Specific attention was devoted to contextual factors that might have had an influence on the two local partnerships. The information generated from the program’s documents allowed the refinement of the study question, in consultation with a number of the program’s stakeholders. Institutional ethics approval for the study was obtained from the St. Mary’s Hospital Centre Research Ethics Committee (No. SMHC-13-30C) on 10 August 2016.

### 2.3. Phase I: Qualitative Data Collection and Analysis

The longitudinal case study of the two partnerships, the Primary Care Connection Partnership and the Community Health Resources Partnership, was conducted between August 2016 and September 2018. This phase entailed non-participant observation of the partnerships’ meetings and semi-structured, in-depth interviews with a sample of the partnerships’ stakeholders. The longitudinal aspect allowed capturing the main developments in the life of the partnerships over time. 

The non-participant observation of meetings focused on the behaviours of partners and interactions among them. The first author conducted the observation of 11 Primary Care Connection Partnership and three Community Health Resources Partnership meetings. Specific attention was devoted to observing how the agreed upon processes common to all partnerships were implemented and to identifying any new emergent processes influencing the partnerships. The semi-structured interviews offered emic (i.e., through the lens of the participants) insights into partnership processes and helped to identify partnership elements that were relevant to the subsequent, quantitative phase. The first author conducted nine interviews with Primary Care Connection Partnership stakeholders and seven interviews with Community Health Resources Partnership stakeholders. Interview participants were asked to report on their experiences within their respective partnerships from the beginning of their involvement until and including the period when the interview was conducted. 

Interview candidates were purposefully selected [64] on the basis of their roles, and the nature and duration of their engagement with the local partnership. Representatives of each of the stakeholder group (i.e., researchers, research program coordinators, policy makers, clinicians, and organizational representatives/patient partners) were invited to participate in the interviews. Attempts were made to ensure a mix of seasoned and new participants. In addition, attempts were made to invite stakeholders demonstrating both high and low levels of engagement, based on prior observation of meetings. 

Candidates selected for interviews were initially invited to participate via blinded group e-mails. Follow up was undertaken in person at the end of partnership meetings, and over e-mail sent directly to the candidates. The duration of interviews was approximately one hour, and they were conducted either in person or over the telephone. The interviews were planned according to existing recommendations on conducting individual interviews in health research [64,65]. The interviews contained predominantly open-ended questions and time was factored in for other questions which arose from the conversation. The questions asked about partnership synergy were inspired by Jones and Barry [58] and Weiss et al. [50]. All interviews were transcribed verbatim. The interview transcripts were not returned to participants for comments. 

A hybrid deductive–inductive approach to framework analysis was used to identify patterns within the data [66,67]. The initial coding scheme was developed to reflect the concepts from the partnership synergy framework, including complementarity of skill, experience and work sharing. Words, sentences or paragraphs from transcripts were extracted into pre-determined codes with new codes emerging from the data. The final codes were grouped along the dimensions of partnership synergy and the five families of factors likely to foster partnership synergy: partner characteristics, relationships among partners, partnership characteristics, resources and external context [8]. The data management, coding and analysis were performed using NVivo 12 qualitative analysis software [68]. The transcripts were analyzed by the first author (EL), but the coding was verified by another qualitative researcher (CS). Emerging findings were discussed at regular team meetings. Phase I resulted in a thick description of the two partnerships as well as on the processes that were employed to enhance the partnerships.

### 2.4. Building on Qualitative Findings

In line with the exploratory sequential design, the qualitative findings were then used to adapt the PSAT partnership self-assessment instrument by using qualitative codes and themes to select the domains and concepts for inclusion in the quantitative phase [69] (see Supplementary Materials Table S1). We selected PSAT scales and items that corresponded to the qualitative codes from the case study and excluded scales and items that were deemed non-relevant. We retained the following PSAT scales: Decision-Making (four items); Leadership (11 items); Administration and Management (11 items); Non-financial Resources (six items); Financial and Other Resources (three items); and Resource Utilization (three items, referred to as “Efficiency” in the PSAT). We also retained questions about perceived Benefits (11 items) and Drawbacks (five items) of participation to stakeholders’ respective organizations and an overall assessment of the benefits compared to the drawbacks (1 item). 

We added elements that emerged in the qualitative data but were not part of the 2001 version of the PSAT: new proposed sub-scales for Communication (three items) and External Environment (two items). The case studies spoke to the importance of communication as an integral dimension of partnership work and the critical role of contextual influences. We also added one question to assess the extent to which the goal of developing a meaningful partnership had been achieved as a measure of partnership effectiveness. We supplemented synergy items from the PSAT with synergy items from the Partner Questionnaire [58] to capture more information on partnership processes. Partnership synergy was assessed using two sub-scales: (1) the adapted Partnership Synergy Processes sub-scale incorporating five items from the eight-item synergy scale developed by Jones and Barry [58]; and (2) the adapted Partnership Synergy Outcomes sub-scale retaining two items from the nine-item synergy scale by Weiss et al. [50]. The items from these original scales were selected based upon the relevance to the types of partnerships highlighted throughout the qualitative inquiry, and with a view to reduce participant burden. Finally, the observation data and responses to qualitative interviews guided the choice of language that was used to elicit descriptive information about the stakeholder (type, role in the organization, length of time in the partnership and frequency of engagement).

### 2.5. Phase II: Quantitative Data Collection and Analysis 

Phase II entailed a cross-sectional survey design. The criteria for partnership inclusion in this phase were: (a) the partnerships had at least five active academic and non-academic partners; (b) the partners had continually and collaboratively worked together to develop and modify strategies in order to achieve their goals; (c) the partners had begun to implement their plans. We distributed a self-administered questionnaire to a census sample of all multiple stakeholders within five of the six IMPACT local partnerships. All stakeholders in five of the six IMPACT local partnerships, who were active in the partnerships at the time of administration, were considered eligible and were invited to participate. The sixth partnership did not meet our inclusion criteria. Our final sample included 54 partnership stakeholders representing five IMPACT local partnerships (a response rate of 90%). The questionnaire captured the subjective input from stakeholders, reflecting their assessment of respective partnerships as separate entities, and not their individual experiences. Both paper- and web-based questionnaires were offered, in either English or French, with multiple contacts to maximize the response rate [70]. The electronic questionnaire was hosted on the *Qualtrics* [71] online survey platform. 

The scores for each sub-scale were derived through unweighted means and medians of all the component items; scores for a respondent were calculated only if at least 50% of the items in the sub-scale had been completed. We used Spearman correlation coefficients to estimate the correlation between items within purported sub-scales, and between sub-scales, specifically partnership processes and partnership synergy. We examined internal consistency of the purported sub-scales by calculating the Cronbach’s Alpha.

At the partnership level, the score for each sub-scale was calculated by aggregating the sub-scale scores of the component member respondents within each partnership. For partnership synergy, the two sub-scales of Partnership Synergy Processes and Partnership Synergy Outcomes were analyzed separately at the level of individual respondents. However, given that they were highly correlated with each other and demonstrated similar correlations with partnership processes, both sub-scales were combined at the level of the partnerships to provide a single score of (total) Partnership Synergy. We used the non-parametric Kruskal–Wallis test to determine if we were able to detect statistically significant differences in sub-scale scores between any of the partnerships. The ranks of the scores were also derived. SPSS 23 for Windows [72] was used for data analysis. 

Parallel to this study, an independent longitudinal qualitative developmental evaluation of the partnerships was undertaken to guide partnership development. The evaluation was designed and led by two of our authors (CS and VL), who subsequently ranked the five partnerships that were the focus of this study, based on the operational definitions and item content of the following sub-scales: Partnership Synergy, Communication, Decision-Making, Problem-Solving, Resource Utilization, and External Environment. We then compared the qualitative and quantitative ranks.

## 3. Results

### 3.1. Study Participants

Study participants represented a range of organizational expertise (see Table 2). The stakeholders within each partnership included a mix of decision makers, primary care physicians, health care managers, academic representatives (researchers and research coordinators, including partnership coordinators), members of vulnerable populations, and stakeholders from community-based organizations providing services to the vulnerable population. Among interview respondents, academic representatives and decision makers constituted the largest two groups (*n* = 10, 63%). Among survey respondents, academic representatives constituted the largest single group of stakeholders in each partnership. Interview participants and survey respondents were predominantly female (interviews: *n* = 13, 81%, survey: *n* = 42, 78%). Of note, 15 stakeholders participated in both the interviews and the survey.

### 3.2. Qualitative Findings

#### 3.2.1. Partnership Assets

Our qualitative investigation revealed that both partnerships had a variety of tangible and intangible assets. These assets were either acquired or generated as a result of working in partnership. 

##### Partnership Acquired Assets 

The main acquired intangible assets in both partnerships were considered by participants to be the resources brought into the partnership by the multiple stakeholders who joined the partnership in order to tackle a complex, multi-faceted issue of common concern—accessibility to PHC for vulnerable populations: “*I think that it’s well organized, and it includes health care utilizers as well, so that’s important, decision makers, researchers and clinicians. So, I think it’s, the structure is aligned well with the area of the study*” (Policy maker). 

These stakeholders contributed unique perspectives and skills, information and connections to a broader set of stakeholders and health systems exerting influence over the partnerships. The medical practitioners shared their experiences of dealing with vulnerable patients and identified health system opportunities and constrains to accommodate new vulnerable patients and to improve services provided to them. The academic team shared relevant research evidence, facilitated the overall work of partnerships and served as an interface with funding bodies and the larger IMPACT program. The research coordinators in particular supported the various components of the interventions, including communicating with stakeholders, organizing partnership activities, facilitating meetings, proactively conducting outreach and gathering and synthesizing information. Patient partners brought the lived experience point of view. Community organizational representatives shared insights into the challenges experienced by the target populations and available community services and helped the partnerships to align project activities with the priorities and capabilities of organizations serving the target populations. Finally, decision makers and health planners served as a bridge between researchers and policy-making, ensuring that research activities aligned with and responded to health policy priorities and capabilities and, conversely, that health authorities were appraised of research evidence relevant to the project. These unique perspectives and insights were deemed to be complementary in that they allowed for the exploration of issues of access from various angles, to obtain timely information in order to make necessary adaptations to the intervention models, and to attract additional resources. The partnership membership was not static, it evolved as the work of the partnerships progressed, reflecting natural stakeholder attrition and the need to attract additional expertise and resources.

The acquired tangible assets included financial resources obtained through the IMPACT research program’s grant funding, space to meet, and information and other project support resources. The partnerships were part of the larger IMPACT research program that received funding in Canada and Australia. This funding covered the partnerships’ coordinating infrastructure/support for research such as data collection, including the partnership coordinator position at each site, as well as the evaluation. Partnership stakeholders, other than research coordinators, were not remunerated for the time spent on partnership activities. However, expenses related to attending partnership face-to-face meetings, disseminating results and promoting the program were covered for most stakeholders. Partnership management activities were carried out by the research teams located at their respective research entities. Face-to-face partnership meetings were organized either at these research entities or at nearby locations, including partner universities, affiliated hospitals, and participating community organizations. Other tangible assets included information resources such as computer software.

##### Partnership Generated Assets 

The generated intangible assets included the skills and relationships that the partnerships had invested in and the learning that transpired in the course of partnership work. The partnerships provided several stakeholders with educational and capacity-building opportunities. The coordinators in each partnership were critical resources who offered ongoing support for partnership activities. Given that working in partnership required skills that were different from those employed in the typical running of research projects, the partnerships made strategic financial investments into acquiring these new skills. Instead of outsourcing certain partnership-related tasks, the partnerships built capacity in-house through training. Both partnerships invested in training partnership coordinators in group process facilitation techniques and then provided them with opportunities to facilitate partnership meetings. Some coordinators highlighted the value of experiential learning:

So, I’ve learned a lot from a research perspective from the research team, and from the LIP Core Team more specifically around just community development and how that works and participatory action research and making sure that everybody has a voice and who needs to be at the table, so that has been really rich, because I knew very little about that when I came on (Research coordinator).

In addition, one of the two partnerships provided its patient partners with opportunities to attend relevant patient engagement training. These training and capacity-building investments were not only part of incentive management, but also benefitted the partnerships directly through enhanced skills and knowledge that strengthened the partnerships. 

Another important generated intangible asset was the collaborative relationships that were formed in the course of partnership work. While some stakeholders had a history of collaborative working relationships, relationships with other stakeholders had to be forged. Each partnership made intentional efforts to strengthen relationships within the respective partnership and with external stakeholders who were critical to partnership efforts. Relationships among some non-academic stakeholders developed more organically as a result of interactions during partnership meetings. These positive relationships benefitted the partnerships by stimulating more open conversations and by contributing to faster and deeper decision-making and enhanced project ownership.

The generated tangible assets included the resources identified to implement and sustain the interventions developed by partnerships. In the absence of funding for intervention implementation under the IMPACT research program’s grant funding, each partnership was required to mobilize adequate local resources to respond to regional access needs and to maintain interventions beyond the life of the IMPACT research funding. Both partnerships developed low-cost lay navigator approaches to addressing the access needs of the target populations. In order to sustain the interventions, both worked towards integrating the interventions into existing health system organizational structures, aligning the proposed models with priority health system initiatives. The Community Health Resources Partnership succeeded at securing additional funding for the initiative, which extended the project beyond IMPACT. The additional funding covered a randomized controlled trial to test the effectiveness of the model that the partnership had developed. 

#### 3.2.2. Partnership Enabling Processes

Both partnerships that were part of our qualitative study [62] employed specific processes to activate the above-mentioned assets. The following main categories of processes emerged from our data: (1) resource management; (2) leadership; (3) administration and management; (4) communication; (5) decision-making; (6) adapting to the evolving context.

##### Resource Management

Stakeholder engagement in the two partnerships occurred in a variety of ways. Both partnerships began with deliberative fora involving a broad range of stakeholders to learn about community needs around PHC access, the relevant community organizations and available resources to support interventions. As partnership work progressed, the Primary Care Connection Partnership involved stakeholders in multiple aspects of the research process, with some non-academic stakeholders fulfilling tasks outside the partnership’s face-to-face meetings. Conversely, the Community Health Resources Partnership took a more traditional academic approach to collaboration with stakeholders—a research advisory approach, with limited contribution of non-academic stakeholders outside partnership meetings. Both partnerships used regular face-to-face meetings to openly discuss project progress, create a shared understanding of the project and engage in iterative collaborative learning: “*I feel like there’s lots of opportunity to share what’s on our minds, to express ourselves. I feel that it’s a very open to dialogue type of meeting.*” (Representative of a community-based service organization); “*I think there is a lot of discussion […], we have the freedom to give our opinion, to discuss. I think it is very appropriate*” (Family physician).

The research teams spearheading the initiative capitalized upon the various strengths and perspectives of stakeholders by providing sufficient time to discuss pressing issues, soliciting input from all stakeholders and offering stakeholders different mechanisms to contribute (e.g., large group discussions and small break-out sessions). Participants who could not attend meetings were appraised of what was discussed during meetings and provided with opportunities to contribute electronically. While interview respondents recognized the inherent power inequalities in the partnerships due to the fact that the research teams controlled grant resources and had more to lose if the partnerships did not achieve stated objectives, they acknowledged that the research teams made efforts to address barriers to equitable participation. Examples included the strategic choice of locations for partnership meetings, transportation fees coverage and approaches to elicit input from linguistic minority participants. Both partnerships experienced difficulty with the engagement of community-based stakeholders representing the target populations; however, after they became involved, one Community Health Resources Partnership patient partner stated: “*[…] it’s a very nice invitation to have people who are not professionals, who are not involved in that kind of world, to be invited in and be allowed to give an opinion or thought or idea. I think it really empowers people*” (Patient partner).

##### Leadership

Both partnerships were largely driven by the research teams that initiated the partnerships. The leadership approaches in the two partnerships differed. The Primary Care Connection Partnership leveraged the power of leadership distributed among academic and non-academic stakeholders with different stakeholders taking responsibility for various components of the work of the partnership. In the Community Health Resources Partnership, the leadership was centralized within the research team. However, interview participants reported that the research team seemed genuinely interested in hearing from all stakeholders and made efforts to check in with various groups around the partnership table: “*[…] there is a very open kind of environment there and, frankly, and you can tell that the research team really wants to hear what people have to say and so they act […] and they ask pointed questions*” (Family physician). 

Despite these differences, there were a number of common leadership processes. Within the research teams, both partnerships had formal (i.e., academic investigators and co-investigators) and informal (i.e., partnership coordinators) leaders knowledgeable about the context and skilled at mobilizing the various perspectives of stakeholders and forging common ground. The leaders did not possess all of the requisite partnership-related knowledge and skills at the outset but rather continuously learned from best practices in partnership development. The leaders were transparent about their own gaps in knowledge and eagerly welcomed input from different stakeholders. This openness contributed to creating an atmosphere of trust where differences of opinion could be voiced. 

##### Administration and Management

The research teams, comprising academic principal investigators, co-investigators and research coordinators, were responsible for the overall management of the partnerships. Each team had dedicated (i.e., full-time) and part-time research staff supporting the work of the partnerships. The scope of the research teams’ activities was broad. They were responsible for recruiting partnership members; managing information; coordinating communication among partners and with outside entities; facilitating the selection, adaptation and implementation of interventions at the local level; evaluating the effectiveness and potential for scalability of interventions. Both research teams performed extensive field work, gathering relevant data and promoting the interventions to external parties. The research teams also organized regular meetings of their respective partnerships. This included organizing meeting logistics, facilitating meetings and summarizing discussions in the form of minutes: “*They seem to just be very effective and very planned and organized. So, I’m learning from seeing how a well-organized meeting unfolds*” (Representative of a community-based service organization). 

The research teams in both partnerships employed adaptive management approaches to support iterative decision-making and facilitated the ability of partners to contribute meaningfully: “*We share, and then the research team stimulates the discussion between different territories, how can we help you, what can we do to succeed. […] In addition, the research team is really glued to the team here to see if something is not working well*” (Policy maker). In addition, the Community Health Resources Partnership conducted meeting evaluations at the end of each partnership meeting and made timely adaptations to how the meetings were run and the type of information provided at subsequent meetings. 

##### Communication

Both partnerships had open and multidirectional channels of communication to communicate internally with stakeholders within the partnership:

Yeah, we get updates, and people are communicating with us. We know that we’re part of the team, we know that we’re being informed, invited to meetings long ahead of time, so there’s lots of opportunity, you know, it’s not a last-minute invitation. So yeah, so those processes are reassuring to see that it’s well run and things are happening in a timely manner (Policy maker).

In-person communication with all stakeholders was mostly confined to regular partnership face-to-face meetings. The research teams synthesized relevant partnership-related information and brought it in condensed verbal and written formats to the attention of the partnership stakeholders. Outside of the meetings, regular contact was supported by partnership coordinators via electronic communication. These exchanges included minutes of meetings, meeting agendas and brief summaries of key issues upon which decisions would have to be made. In both cases, participants highlighted the importance of learning loops and having a variety of ways of soliciting input from partners. Learning loops involved requesting feedback from participants during meetings around issues relating to the project and being transparent about how this input was subsequently incorporated including being explicit about the reasons why suggestions could not be incorporated. 

Partnership external communications were aimed at increasing support for partnership interventions, recruiting medical practices that would be part of the interventions and disseminating information about partnership activities and achievements more broadly to the program’s funders and the broader academic and medical community. External communication occurred during conferences in the form of conference posters, oral presentations and workshops as well as during scheduled meetings with relevant external entities. In addition, the Community Health Resources Partnership produced a periodic newsletter regarding project activities with a broader community reach.

##### Decision-Making

The Primary Care Connection Partnership stakeholders reported that the decision-making process was transparent and inclusive. Main decisions pertaining to the project were taken during face-to-face partnership meetings, by vote, on the basis of information compiled by the research team and following extensive stakeholder consultations: “*Decisions, I think the fact that you go to a vote [...] to decide how to make a change, I think that’s good. They take all opinions into account before making a major decision*” (Family physician). This was particularly apparent in relation to adapting the intervention to its evolving context. Conversely, in the Community Health Resources Partnership the decision-making power was centralized within the research team, which was consistent with the advisory nature of the partnership.

##### Contextual Adaptation

The activities of both partnerships were unfolding within the context of significant health care system reforms in their respective provinces. While both partnerships had to make adaptations to the interventions to respond to evolving contextual opportunities and threats, the extent of contextual impact and adaptation was far greater in the case of the Primary Care Connection Partnership: “*Of course, the thing that doesn’t change is the changes in the [health] networks. But this is quite a major change.*” (Policy maker). The partnership made several adaptations to the intervention, including its structure, delivery strategy and personnel resources. The situational analysis involved harnessing the knowledge of multiple stakeholders, instead of using formal environmental scanning analysis approaches. The active engagement in the partnership of policy makers and health system planners was critical in this respect, in that it contributed to an in-depth understanding of the context. Having stakeholders around the table with medium to high level of authority in their respective organizations allowed adaptations to be implemented in a timely manner. 

#### 3.2.3. Partnership Synergy

Partnership synergy manifested itself in the combination of the complementary skills and unique perspectives of the partners: “I think it’s a really good mix of people, and you can hear it in the discussion. The very different points of view, and they all complement each other very well” (Representative of a community-based service organization).

I honestly don’t think that there’s any other way to do it, because it’s in primary care, and primary care is incredibly complex, there are so many players involved […] incredibly complex problems and challenges, you know, particularly more so for the populations we are interested in, vulnerable populations […] plus things are changing all the time, funding is changing and so on, so we always have to situate our project in a larger context. If we didn’t have those other people at the table, how would we know what’s going on […] (Research coordinator).

Discussing the added value of partnership work some participants described the alignment of efforts of partnership stakeholders and the richness and integrative nature of collaborative work: “*These [partnership] tables are an example of integration. […] We become more integrated and stronger, and there is a certain level of coherence between us. It has to be like this*” (Policy maker, emphasis by the participant). “*It is very rich. Not everyone has the same reality, and we inspire each other. In understanding the point of view of the other, we advance the discussion*” (Policy maker).

[…] Magic happens when you get people who are going in the same direction. So, it’s just about … it’s analogous to integrating the health care system. If you have a group that is fragmented and they’re doing their own thing separately the result obviously can’t match the result that can happen when you’re working together. So, it’s all about integrating the effort. And so, it has to be better than just doing it on your own (Policy maker).

Partnership synergy was also apparent in a variety of anticipated and actual benefits reported by stakeholders, stemming from their participation in the partnership, and in their sustained commitment to the partnerships: “*And there are different important constituents, it is more than a political representation of organizations, people who have continued to participate because they believe in it*” (Research coordinator).

Participants described more benefits related to their respective organizations than personal benefits, highlighting a fit between the project’s objectives and the organizational priorities of the entities they represented. The mutually beneficial nature of the partnership was reported by participants who stressed mutual and personal learning and satisfaction that stemmed from their involvement in the project: “*The researchers are learning from each other*” (Research coordinator).

For me, this is completely new to me to be part of this type of project, so it’s a learning experience that I’m enjoying tremendously. So, it’s definitely good for my personal development to be part of this, I’m kinda honored to be part of this […] (Representative of a community-based service organization).

So, to be able to be part of the project which I think that they had a great idea, it’s really smart, and I felt really glad to be part of that. You know because I feel like that’s a good project, this is very helpful, this is a very, you know, significant issue for people. And to be able to be part of maybe, you know, exploring why it’s a problem and offering my insights, I’m very excited to be able to do that (Patient partner).

Partnership synergy was also evident in innovative ideas and new solutions to the presenting challenges: “We are experimenting with innovative practices. Teamwork goes further. Alone we may be faster. Together we go less quickly, but we have good results, and likely sustainable results” (Policy maker).

There was evidence of partnership synergy in the ability of both partnerships to pull resources and problem-solve, to sustain interventions over time, despite contextual challenges and funding gaps:

When the reform arrived, everything changed. We had to redevelop relationships, see who was going to be at the tables, did we want to keep our original territories or expand to the territories of the new [health authority]. […] We then managed to identify a problem, we identified an intervention, which we put in place, which does not work, which we have adapted and are in the process of putting back in place (Research coordinator).

Partnership synergy also manifested itself in the positive partnership atmosphere, in the feeling of unity, and the relationships that were forged in the process of working together: “*[…] It’s a good trusting environment. People are happy to speak up and say what they need to say. Everybody seems to be happy to be involved*” (Policy maker). “*I usually see it as we all come together, sort of. I don’t feel […] that there’s some difference between anyone, I feel like we’re equally sitting at the same table, like this one single group*” (Patient partner).

Other participants highlighted the “feeling of belonging” (Family physician) and increased project ownership: “The commitment to the project is higher when you have built it together […] When you have done it in collaboration, it is closer to your heart and I think that this is one of the advantages” (Research coordinator).

### 3.3. Quantitative Results

Table 3 displays correlations among partnership assets, the various categories of partnership processes and partnership synergy as assessed using Spearman’s rank correlation coefficient. We used the following cut-offs for interpreting the scores: 0.10—weak correlation; 0.30—moderate; 0.50—strong (29). The results indicate that partnership synergy outcomes were strongly associated with decision-making, leadership and administration and management, while partnership synergy processes were strongly associated with decision-making, leadership and non-financial resources. Total partnership synergy was strongly associated with decision-making, leadership, administration and management, non-financial resources and resource utilization. 

We subsequently analyzed the scores for partnership synergy outcomes, partnership synergy processes and total partnership synergy in each of the five partnerships. Our one-way analysis of variance was suggestive of statistically significant differences between at least two of the medians of partnership synergy processes (*p* = 0.09) and (total) partnership synergy (*p* = 0.07). The differences in medians were statistically significant between the following partnerships: Primary Care Connection–Service Linkage, Community Outreach–Community Health Resources, Community Outreach–Diabetes Self-Management and Community Outreach–Service Linkage (in decreasing order of magnitude of difference). No difference was observed when sub-scales were analyzed separately. 

We then ranked the five partnerships on the basis of their aggregate scores for partnership synergy outcomes and processes and compared our results with independent, qualitative rankings of partnerships performed by content reviewers. The quantitative and qualitative rankings were completely coherent for partnership synergy outcomes, showing that partnership synergy was highest in the Community Outreach Partnership and lowest in the Service Linkage Partnership. However, there was a slight difference in the rankings of two partnerships by partnership synergy processes, namely, the Community Health Resources Partnership and the Diabetes Self-Management Partnership.

**Figure 2:**
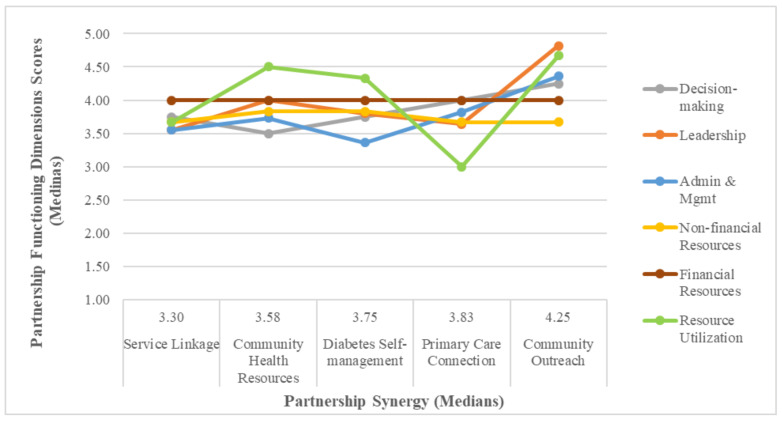
Comparison of values of partnership assets, processes and total partnership synergy by partnership (*n* = 5) (reproduced from [63]).

Our quantitative analysis revealed that Partnership Synergy Outcomes and Partnership Synergy Processes sub-scales were strongly correlated with each other (*r* = 0.61), and there were few differences in how each of them correlated with the different dimensions of partnership work (see Table 3). We consequently chose to collapse the two synergy sub-scales together to a single Partnership Synergy scale (Partnership Synergy) for the analysis of associations at the partnership level. We also aggregated the scores at the partnership level for partnership assets and the following categories of partnership processes: non-financial resources, financial resources, resource utilization, decision-making, leadership, and administration and management (see Figure 2). Our newly developed sub-scales of Communication and External Environment did not perform well metrically, on assumptions of item-convergent validity, item-discriminant validity, and internal consistency. Therefore, we excluded these sub-scales from partnership-level analysis. The items related to the domains of communication, external environment, and benefits and drawbacks were analyzed independently for descriptive purposes only.

We hypothesized that partnerships exhibiting higher partnership synergy would on average achieve higher scores for partnership assets and processes. On the other hand, partnerships exhibiting lower partnership synergy would on average achieve lower scores across various categories of assets and processes. Our data (Figure 2) indicate that stakeholders in the Community Outreach Partnership did report on average the highest scores on all categories of partnership assets and processes, with the exception of non-financial resources, and that the Service Linkage Partnership stakeholders reported on average the lowest or second lowest scores for all categories of assets and processes. Our within partnership correlation analyses to investigate relationships between partnership assets, processes and synergy did not yield conclusive results due to the small number of observations in each partnership. 

Figure 2 presents the complex picture of partnership work. All partnerships display variable scores, higher in some categories and lower in others. The figure illustrates that the composite elements constituting the performance of each partnership interact in a multitude of ways, and that there is no single path towards achieving a partnership synergy rank. We also observed that administration and management and decision-making processes appeared to be more critical to establishing the total partnership synergy rank than other processes.

Irrespective of the variations in scores for different partnership assets and processes, the partnership-level scores for achieving the overall goal of developing a meaningful partnership were largely consistent with partnership-level partnership synergy scores. The majority of respondents in all partnerships reported that a meaningful partnership had been achieved leading to an uninformative median of four across all partnerships. However, as shown in Table 4, there was considerable variation within the partnerships that was reflected in the means and standard deviations. The metric that corresponded best to both partnership synergy scores and the qualitative ranking was the percentage endorsing that the partnership goal was achieved *very well* or *extremely well*.

### 3.4. Integration of Qualitative Findings and Quantitative Results

Table 5 below provides the comparison of our qualitative findings and quantitative results. 

## 4. Discussion

This empirical exploratory study provided insights into complex and dynamic partnership processes and into how partnership assets are activated to produce partnership synergy. The Partnership Synergy framework posits that “[…] synergy is the degree to which the partnership combines the complementary strengths, perspectives, values and resources of all partners in the search for better solutions and is generally regarded as a product of a partnership” [6]. We have expanded this definition to incorporate the contribution of enabling (facilitating) processes in combining the tangible and intangible assets that are the inputs of the partnership. It is the combined effect of the inputs and enabling processes that confers advantages over the work of individual agents. Our first key finding of this study relates to the association between partnership synergy, tangible and intangible partnership assets and enabling collaborative processes. While enabling collaborative processes could be both operational and interpersonal in nature [5], this study focused primarily on operational facilitators and system-level processes geared towards making ongoing adaptations to the evolving context. We assessed the role of facilitative leadership, supportive administration and management approaches, mechanisms of engaging partners and capitalizing on partners’ resources, communication, and collaborative approaches to managing change. Our results (Table 3) indicate that partnership synergy was associated with partnership dimensions of leadership, administration and management, decision-making, and the ability of partnerships to optimize the involvement of its partners (referred to as “Efficiency” in the Partnership Synergy framework and referred to as “Resource Utilization” in our study). It was also associated with the sufficiency of non-financial resources. We could not perform correlation analyses to investigate relationships between partnership assets, processes and synergy at the partnership level due to the limited number of observations in each partnership. However, our comparison of values for partnership processes, the sufficiency of partnership assets and total partnership synergy by partnership (Figure 2) indicates that among the partnership enabling processes investigated in our study administration and management and decision-making seem to be more critical to determining the achievement of partnership synergy. Our study findings also support the importance of recruiting partnership stakeholders with varied but complementary expertise and of benefits to the stakeholders’ respective organizations. 

Our Communication and External Environment sub-scales did not perform well metrically [63]. However, the Spearman rank correlations between all three communication items and partnership synergy were moderate, supporting our qualitative conclusion that communication was an important dimension of partnership functioning. Conversely, our newly developed sub-scale of External Environment was not correlated with partnership synergy nor any other dimension of partnership functioning. The lack of correlation may be explained by the limitations of the sub-scale used. Alternatively, the lack of correlation in our study may be explained by the fact that the interface between a partnership and its context is complex and difficult to capture with standardized tools. Our qualitative investigation revealed that recognizing and dealing with changes in partnership context were important to achieve partnership synergy [62]. In addition, the synergy generated in both partnerships under the qualitative investigation facilitated adaptation to the challenging contextual circumstances and allowed the partnerships to continue. How these variables will interact under different types of contextual threats, such as the global COVID-19 pandemic, needs to be further examined. Given the importance of face-to-face interactions highlighted by our study participants, generating partnership synergy may be more challenging when moving to virtual forms of communication and interactions necessitated by the pandemic. Moreover, in our study, the nucleus of each partnership, which included the research team and a number of key non-academic stakeholders, remained consistent over time, while new members were invited to join based on the evolution of the projects and the need to attract specific expertise and additional resources at different points in time. Maintaining the consistent continuous core of members who provide continuity and keep the collaboration going may be impossible to achieve when there are sudden changes in priorities at all levels of the system and when key personnel and other resources need to be re-deployed. Changes in government priorities and policies may lead to a redefinition of partnership goals or may mean that the specific concerns driving the partnership have ceased to exist altogether [73]. However, the synergy generated by a well-functioning partnership that is already underway may help partners to quickly mobilize their strengths and reposition the partnership to tackle new priorities and vulnerabilities exposed by the pandemic. 

Our findings are consistent with prior research that has assessed relationships among different partnership functioning factors and partnership synergy [6,50]. This research has analyzed the associations between partnership functioning factors, partnership synergy and the following partnership outcomes: effectiveness in the delivery of chronic illness care [56], sustainability of innovative programs in community care [55] and perceived partnership sustainability and perceived community outcomes in sport-for-health partnerships [74]. Applying similar measures of partnership functioning and partnership synergy, studies demonstrated that partnership synergy was associated with the effectiveness of partnership leadership [6,50,55], partnership efficiency [6,50], the effectiveness of administration and management [50] and the sufficiency of non-financial resources [50,56]. It has also been established that partnership synergy acted as a mediator between partnership functioning and partnership outcomes (e.g., [56]). In our study, partnership synergy emerged as an intermediate outcome of partnership functioning, before the intended outcomes related to PHC access could be assessed. Given the stage at which the partnerships were studied, and that identifying causal pathways was beyond the scope of this research, the study did not incorporate a separate effectiveness assessment of the intended outcomes. It therefore could not demonstrate definitively whether higher levels of partnership synergy precipitated better achievement of intended outcomes. However, the majority of survey respondents in all studied partnerships reported having developed a meaningful partnership that achieved both added value and benefits that largely outweighed the drawbacks of participation. In addition, a recent parallel study of IMPACT produced evidence that the intended goals of the program were largely met through partnerships [75]. The study by Spooner et al. [75] found that all IMPACT sites observed changes in patient abilities to access PHC and in provider capabilities to address the health care needs of vulnerable populations, even in interventions where there was no activity intentionally targeting provider behaviors. These findings are indicative that the partnerships were effective in reaching the intended outcomes. 

Our comparison of qualitative and quantitative findings revealed additional nuances in relation to the type of leadership that was employed in the partnerships that were the focus of our qualitative investigation. Prior studies have highlighted the critical importance of leadership to partnership synergy [6]. The study by Weiss et al. (2002), using the same PSAT leadership scale, found that leadership was the dimension that was most closely associated with partnership synergy. The type of leadership that partnerships necessitate is facilitative and “boundary-spanning”—enabling those in charge to appreciate and bridge the diverse perspectives of various stakeholders [8]. It needs to be shared formally and informally among stakeholders, even though there may be one “lead” organization [6]. This type of leadership, referred to with a variety of terms including “plural”, “collective” or “distributed” leadership or simply DL, is located at the other end of the spectrum from individual leadership [76,77,78]. It refers to a “collective phenomenon that is distributed or shared among different people, potentially fluid, and constructed in interaction” [77]. The partnership that fit this definition based on our qualitative findings did not achieve the expected higher score for leadership. This observation suggests that while the leadership sub-scale used in our study tapped into its facilitative and boundary spanning nature, it might not have reflected its distributed power. Given the centrality of leadership to multi-stakeholder partnerships in general and partnership synergy in particular, future research should explore how the PSAT leadership scale could be amended to reflect DL. Taking into consideration the temporal and contingent nature of leadership, whereby certain situations and earlier partnership phases will require more centralized forms of leadership [76,79], it might not be possible to develop an all-encompassing leadership scale. This challenge may be addressed through the assessment of the various partnership components longitudinally and with the use of mixed methods, allowing the collection of different types of data.

Our qualitative and quantitative results were also less consistent in relation to the dimension of financial resources. Our qualitative investigation demonstrated the critical importance of the funded coordinating infrastructure and that partnerships were capable of mobilizing adequate tangible resources to sustain service interventions. Our quantitative results revealed a high score for financial resources across all studied partnerships but a low correlation with most other dimensions of partnership functioning and Partnership Synergy Outcomes. This low correlation suggests that the Financial Resources sub-scale that we used might not have reflected the complexity in the types of tangible resources that were at play in IMPACT partnerships [63]. Alternatively, it may be explained by the fact that some multi-stakeholder health partnerships have few tangible resources. While financial and other capital resources are some of the building blocks sustaining partnership operations and partnership service interventions, the essence of partnership work lies in the production of knowledge and in relationship building. In fact, partnerships can be viewed as “knowledge organizations” that utilize intangible assets, such as the skills and expertise of partners, to convert information into knowledge [53]. While financial and other capital resources are important, non-financial resources seem to be more critical to creating partnership value including the ability of partnerships to make decisions, effective leadership and partnership management and administration. This finding is consistent with resource-based theory that suggests that intangible resources may be more valuable for they are more difficult to acquire and replicate [51].

Our second key finding relates to the contingent nature of partnership work and organizing to operationalize the partnership. We hypothesized that partnerships exhibiting higher partnership synergy would, on average, report higher levels of partnership functioning across various dimensions. On the other hand, partnerships exhibiting lower partnership synergy would, on average, report lower levels of partnership functioning across various dimensions. Our results indicate that the partnership with the highest total partnership synergy score achieved, on average, the highest scores for partnership functioning on most studied dimensions. Conversely, the partnership with the lowest total partnership synergy score achieved, on average, the lowest or second lowest scores for partnership functioning on all dimensions. However, the results for partnerships with middle total partnership synergy scores were more varied. The Primary Care Connection Partnership achieved a high synergy score despite a number of lower scores on some partnership functioning sub-scales. On the other hand, the Community Health Resources Partnership that achieved the second lowest total partnership synergy score performed well on some of the partnership functioning dimensions (such as resource utilization). These findings reinforce contingency theory concepts [80,81], in that they reflect the complexity in partnership work and suggest that there is more than one way of organizing and reaching partnership synergy. They also suggest that there might be certain partnership dimensions that are still lacking from our analysis. Taking into consideration the emphasis that the stakeholders in all five partnerships put on the quality of relationships, our assessment could have benefitted from incorporating measures of interpersonal collaborative processes and the quality of stakeholder relationships including such aspects as trust. In fact, prior research has highlighted relational factors, such as high levels of trust, to be important ingredients in a well-functioning partnership [82,83,84], and trust has been identified as a critical determinant of partnership synergy [6]. 

A third key finding highlights the importance of assessing partnership synergy as an indicator of the health of the partnership. It might not be feasible or necessary to capture all possible dimensions of partnership functioning to be studied or assessed with standardized scales, for partnership synergy amounts to more than the sum of its parts [8,50,85]. Given that partnerships are inherently complex, highly contextual and constantly evolving organic entities, different factors may be more or less relevant depending on the specific circumstances and the goals of a partnership [78,86]. At the start of the IMPACT program, the sites agreed to work with their respective local constituents *in partnership*. As the time progressed each partnership under our investigation evolved in different ways, addressing distinct PHC access needs, applying different ways of engaging stakeholders and developing unique ways of responding to contextual challenges. Therein lies the value of assessing partnership synergy as an important intermediate partnership outcome and a barometer for the health of the partnership. Confining partnership evaluation to assessing performance on individual partnership functioning indicators would provide important but partial information on how a partnership scored vis-à-vis set performance targets or, in comparative research, other partnerships. This partial analysis may lead us to argue that the Primary Care Connection Partnership that achieved lower partnership functioning scores on a number of dimensions did not perform well. However, the high partnership synergy score for this partnership depicts a different picture, pointing to the effectiveness of dynamics and processes and a more “holistic” view of the partnership. We could not assess partnership synergy longitudinally because this study commenced when the partnerships had already achieved maturity. Evaluating partnership synergy, qualitatively and quantitatively, as an evolving indicator of partnership health, at different points in the partnership’s life, would allow the collection of data on other processes that might have contributed to higher (or lower) levels of partnership synergy over time. In addition, observing fluctuations in partnership synergy and partnership functioning dimensions scores may allow researchers to infer the relative importance of these dimensions to synergy.

Initiating and sustaining partnerships is not easy; it is a complex, dynamic and time-consuming process that involves multiple tasks and requires certain vital components [36,46]. This study contributes to the evolving body of knowledge on partnership synergy as a useful framework for studying collaborative ventures and identifies some of the critical requirements for synergistic partnerships. These requirements should be considered in order to determine if working in partnership should and can reasonably be pursued. Though the transferability and generalizability of this study’s findings may be limited due to the small sample size, the findings are likely robust for academic partnerships with partners in the formal health care system. Despite the specificity of partnerships under this investigation, there was enough heterogeneity among them in terms of their contexts, their composition and how the partnerships evolved, suggesting relevance of these findings across various settings. It has been argued in the partnership literature that despite the diversity of partnerships, there are a number of common features influencing their effectiveness, and that the underlying principles behind creating and sustaining effective partnerships are generic [87]. The concepts used in this study in general and the PSAT in particular have demonstrated robustness in assessing the quality of partnership processes and outcomes in multi-stakeholder partnerships in several areas including public health, health promotion and chronic illness care [2,6,56]. The PSAT has also been identified as a valid tool for measuring group processes in interprofessional health and social service partnerships at the front-line service provider group level [2]. However, the evaluation approach and the PSAT will need to be tailored to different partnership types and local conditions. For example, community-driven partnerships with fewer resources, a decentralized partnership structure and more distributed decision-making may need to adapt the Leadership and Administration and Management sub-scales. Alternatively, partnerships may prioritize different aspects for evaluation altogether. The list of variables offered by the Partnership Synergy framework allows partnership practitioners and evaluators to select those relevant to a particular partnership. 

### Limitations

The findings of this research need to be considered in light of its limitations. First, our findings are based on a relatively small sample of partnership stakeholders who were part of a single funded program of research with a specific focus of enhancing accessibility to PHC for vulnerable populations. Caution is warranted when transferring these findings to partnerships operating in different political, resource and health care delivery environments. Second, the statistical power of our quantitative conclusions is limited due to the small sample size. Third, while we made attempts to ensure the collection of a diversity of opinions, given the voluntary nature of engagement and the timing of data collection, such that those who did not see value in the partnerships would have resigned, participants may have provided a more favourable assessment of their respective partnerships. Finally, our analysis only included a number of constructs, based on the selected theoretical frameworks and on our qualitative data. We may not have captured all of the manifestations of partnership synergy, assets and processes. 

## 5. Conclusions

This research was conducted with the aim of understanding partnership processes and how various partnership factors contribute to partnership effectiveness. The partnerships involved stakeholders from different organizations with an interest in implementing organizational solutions that enhance access to primary health care for vulnerable populations. Effectiveness was conceptualized both in terms of the achievement of intended outcomes and the effectiveness of processes (the internal health of the partnership). This research offers several original contributions to the theory and practice of multi-organizational multi-stakeholder health partnerships. First, it demonstrated the applicability of and further refined the theoretical concept of partnership synergy as an indicator of the health of a partnership and an intermediate outcome of working in partnership. Second, the research affirmed the importance of investing in the assessment of partnerships and contributed to the emerging knowledge on how to evaluate them. Third, by demonstrating an association between partnership assets, enabling processes and synergy, this research underlined the operational importance of investing in and paying attention to partnership processes as part of achieving both partnership synergy and the intended outcomes of multi-stakeholder partnerships.

## Figures and Tables

**Table 1:** Overview of characteristics of interventions in six IMPACT * local partnerships (adapted from [34,35]).

Partnership Title	Service Linkage	Community Health Resources	Diabetes Self-Management	Primary Care Connection	Community Outreach	Residential Aged Care
**Target population and access problem**	Vulnerable clients of community-based chronic disease services without a primary care provider.	Primary care patients with complex health and social needs not receiving available community services that would optimize their illness management.	Patients with low health literacy and other social vulnerabilities presenting to general practice with poorly controlled diabetes.	Unattached patients in high deprivation neighbourhoods who have trouble connecting effectively to newly assigned family physicians from centralized wait lists.	Individuals and groups of socially vulnerable populations living in a geographic area with few PHC ** services and high concentration of marginalized populations.	Aged and frail residents of long-term care facilities who have complex chronic health needs.
**Type of vulnerability**	Low socioeconomic status, social isolation due to the fact of geographic distance/public transport, chronic illness and developmental disability.	Socially complex patients, including one of Canada’ linguistic minorities.	Low socioeconomic status, geographic isolation and culturally and linguistically diverse backgrounds.	Low income, unemployment and low social support.	Recent immigrants, Aboriginal people, seniors and homeless persons.	Socially isolated frail elders who rely on others for the provision of care.
**Intervention type**	Brokerage service to link identified patients to one of a panel of volunteer family practitioners.	Training provided to primary care providers to increase their awareness of community health resources; community-based intervention by a lay patient navigator to facilitate access to these resources.	A website that provides information and referral options to support diabetes self-management.	Telephone outreach by lay volunteer navigators to relay access-related and visit preparation information.	Mobile (pop-up) outreach by a variety of PHC and social service providers catering to the needs of attendees, held at different locations.	Training and policy and procedure redesign in participating long-term care facilities to improve the quality, consistency and responsiveness of PHC **.
**Intended consequence**	Successful linkage with a PHC ** practice.	Increased referrals to community health resources and improved access to these services.	Successful affiliation to a coordinating primary care physician and improved diabetes self-management skills.	Successful affiliation to a family physician.	Enduring relationships between PCH and social service providers and the users of mobile pop-up services.	Reduction in preventable hospitalizations and improvements in the delivery and management of care to frail elderly residents.

* IMPACT—Innovative Models Promoting Access-to-Care Transformation; ** PHC—primary health care.

**Table 2:** Study sample characteristics per partnership (*n* = 5) (reproduced from [63].

Partnerships	Total Number of Participants (*n =* 54)	Gender	Mean Length of Time in Partnership	Minimum/Maximum Length of Time in Partnership	Main Role
		Percent Female			
**Service Linkage**	9	78% (7)	3.8 years	2–6 years	Academic representative—4 Decision maker—1 Health care manager—4
**Community Health Resources**	19	68% (13)	2.6 years	1–4 years	Academic representative—5 Community organization representative—3 Decision maker—2 Health care manager—4 Patient representative—3 Primary care physician—2
**Diabetes Self-Management**	7	71% (5)	3.1 years	0.7–5 years	Academic representative—3 Community representative—1 Decision maker—1 Health care manager—1 Primary care physician—1
**Primary Care Connection**	12	83% (10)	3 years	1–5 years	Academic representative—5 Decision maker—1 Health care manager—3 Patient representative—1 Primary care physician—2
**Community Outreach**	8	86% (7)	2.8 years	2.5–5 years	Academic representative—6 Decision maker—1 Health care manager—1

**Table 3:** Spearman rank correlations among partnership synergy outcomes, partnership synergy processes, total partnership synergy and the different partnership assets and processes (*n* = 54). Values shaded in light grey denote weak correlations and in dark grey strong correlations (adapted from [63]).

	Partnership Synergy Processes	Communication	Decision-Making	Leadership	Administration and Management	Non-Financial Resources	Financial Resources	Resource Utilization	External Environment
**Partnership synergy outcomes**	0.61	0.34	0.60	0.69	0.60	0.39	0.18	0.46	0.15
**Partnership synergy processes**		0.44	0.59	0.60	0.48	0.51	0.35	0.45	0.26
**Total partnership synergy**		0.40	0.66	0.74	0.61	0.51	0.28	0.50	0.22
**Communication**			0.47	0.50	0.45	0.38	0.32	0.39	0.10
**Decision-making**				0.65	0.52	0.40	0.07	0.29	0.25
**Leadership**					0.74	0.42	0.13	0.53	−0.08
**Administration and management**						0.42	0.05	0.50	−0.06
**Non-financial resources**							0.56	0.32	−0.21
**Financial resources**								0.28	−0.18
**Resource utilization**									−0.17

**Table 4:** Partnership-level assessment of the extent to which the goal of developing a meaningful partnership was achieved (reproduced from [63]).

Partnership	Not Well at All or Not so Well	Moderately Well	Very Well or Extremely Well	Mean (Standard Deviation)
**Service Linkage (*n* = 9)**	2 (22.2%)	3 (33.3%)	4 (44.4%)	3.22 (1.20)
**Community Health Resources (*n* = 15)**	0	5 (33.3%)	10 (66.7%)	3.87 (0.74)
**Diabetes Self-Management (*n* = 7)**	0	2 (28.6%)	5 (71.4%)	3.86 (0.69)
**Primary Care Connection (*n* = 10)**	1 (10%)	2 (20%)	7 (70%)	3.60 (0.70)
**Community Outreach (*n* = 7)**	0	1 (14.3%)	6 (85.7%)	4.14 (0.69)

**Table 5:** Presentation of qualitative, quantitative and comparative results related to partnership assets, enabling processes and partnership synergy.

Determinants of Partnership Synergy	Phase I Qualitative Findings Based on 14 Meeting Observations and 16 Interviews in Two Multi-Stakeholder Partnerships	Phase II Quantitative Results Based on Survey of 54 Partnership Stakeholders Representing Five Multi-Stakeholder Partnerships	Comparison of Qualitative Findings and Quantitative Results—Comments
**Partnership Assets**			
**Intangible assets—non-financial resources (can be acquired or generated)**	Main acquired intangible assets: Heterogeneous and complementary perspectives, skills and information reflected in partnership composition; Connections to a broader set of stakeholders and influence; Changes in stakeholders brought in new assets as needed for complexity and scope of the task; Stable assets assured by core stakeholders, consistent over time. Generated intangible assets: Training in skills specific to partnership project; Collaborative relationships intentionally built; Collaborative learning evidenced in deeper knowledge and enhanced skills.	Significant intangible assets were reported across all five partnerships. 84% of survey respondents reported that their respective partnerships had *most* or *all* of the needed skills and expertise to work well. 75.9% of survey respondents reported that the skills and unique perspectives of partners complemented each other *often* or *always*. There was a strong association between partnership synergy and non-financial resources (*r*_s_ = 0.51).	Both qualitative and quantitative findings suggest that intangible assets contributed to partnership synergy through having appropriately complementary and heterogeneous skill sets. Heterogeneity and complementarity were achieved by having a dynamic group composition that reflected the critical dimensions of the problem to be addressed and of the context that was likely to affect the work of the partnership.
**Tangible assets—financial and other capital resources**	Research funding facilitated partnership activities; other partners contributed in-kind resources such as time and space to meet and conduct partnership activities. Partners leveraged adequate financial resources to sustain interventions and partnership activities.	The majority of survey respondents (96%) reported the presence of financial and other capital resources across all five partnerships. There was a weak association between partnership synergy and financial and other capital resources (*r*_s_ = 0.28).	Qualitative and quantitative findings are partially consistent, suggesting that the importance of financial resources for partnership synergy related principally to supporting the coordinating infrastructure and a number of partnership activities (such as evaluation and outreach). Non-financial resources seemed to be more critical for partnership synergy than financial resources.
**Partnership Enabling Processes**			
**Asset/resource management**	A variety of engagement mechanisms, consistent with partnership configuration and mandates, to solicit input from stakeholders within and outside the partnerships. Evidence of partnership synergy in the integration of non-financial and financial resources: in optimal and sustained level of commitment to the initiatives for most stakeholders, in collaborative learning relationships, in a variety of reported benefits for stakeholders’ respective organizations, and in the ability of partners to leverage adequate financial resources. Difficulty meaningfully engaging community-based stakeholders from or representing the target population, for both partnerships, and reduced engagement of some academic stakeholders in the Primary Care Connection Partnership.	The majority of survey respondents reported high levels of engagement in partnership activities. The majority of survey respondents reported that their respective partnerships made very *good* or *excellent* use of their time (70%, excluding Primary Care Connection and Service Linkage) and other non-financial resources (67% excluding Primary Care Connection), with the highest rates reported in the partnership with the highest total partnership synergy score (Community Outreach). When asked how well the partners were able to include the views and priorities of the people affected by the partnership’s work, the majority of survey respondents (59.2%) stated *not so well* or *moderately well*. There was a strong association between partnership synergy and resource utilization (*r*_s_ = 0.50). The majority of respondents (78%) stated that the benefits of participating for the organizations that the stakeholders were representing in the partnership *exceeded* or *greatly exceeded* the drawbacks. The partnership with the highest total partnership synergy score reported, on average, the highest score for benefits, and the partnership with the lowest total partnership synergy score—the lowest.	Both qualitative and quantitative results indicate that high levels of stakeholder engagement were important to achieve partnership synergy. The nature of engagement has to be aligned with the function of the partnership and the need to fulfil project objectives, with particular attention to meaningful engagement of end users and addressing disengagement. Benefits related to respective organizations seem to be more critical than personal benefits. Managing incentives, so that benefits outweigh costs, is an important consideration.
**Leadership**	Research teams assumed leadership of processes in both partnerships. Leaders capitalized upon the various strengths and perspectives of stakeholders, contributing to partnership synergy. The Primary Care Connection Partnership leveraged the power of leadership distributed among academic and non-academic stakeholders.	The partnership with the highest score on the partnership synergy scale (4.25) achieved the highest score on the leadership sub-scale (4.82). The results for partnerships with lower partnership synergy scores were more mixed. Primary Care Connection achieved a higher partnership synergy score (3.83) than Community Health Resources (3.58) but a lower score on the leadership sub-scale (3.64 vs. 4.00). There was a strong association between partnership synergy and leadership (*r*_s_ = 0.74).	Qualitative and quantitative results suggest that partnership synergy was facilitated by leadership capable of mobilizing the various perspectives of stakeholders. Qualitative results also highlight the contribution to partnership synergy of more distributed forms of leadership; however, the quantitative results suggest the limitations of the sub-scale in terms of assessing the extent to which leadership was distributed.
**Administration and management**	Research teams, including dedicated partnership resources, responsible for the operational running of the partnerships. Adaptive management approaches, employed by the research teams, supported the work of the partnerships and facilitated the ability of partners to engage meaningfully, contributing to partnership synergy.	There was a strong association between partnership synergy and administration and management (*r*_s_ = 0.61).	Qualitative and quantitative results highlight the importance for partnership synergy of a core infrastructure and adaptive management approaches to support the work of the partnerships and facilitate the ability of partners to contribute meaningfully.
**Communication**	Regular face-to-face meetings and timely and varied communication mechanisms employed by both partnerships. Open and multidirectional channels of communication and learning loops allowed stakeholders to stay informed and engaged, contributing to partnership synergy.	The majority of respondents reported being *adequately* or *always informed* regarding partnership’s activities. Our newly developed Communication sub-scale did not perform well metrically.	Qualitative findings point to the importance of timely and varied communication mechanisms in synergistic partnership learning. However, quantitative results were limited, precluding definitive conclusions regarding the contribution of communication to partnership synergy.
**Decision-Making**	The Primary Care Connection Partnership harnessed the power of distributed decision-making and collaborative problem-solving, contributing to synergy. Stakeholders participated actively in the co-construction of the various aspects of the project, and some non-academic stakeholders fulfilled tasks outside the steering committee meetings. Decision-making power was centralized within the research team in the Community Health Resources Partnership, consistent with the advisory nature of the partnership.	Partnerships with higher scores on the partnership synergy scale achieved higher scores on the decision-making sub-scale. There was a strong association between partnership synergy and decision-making (*r*_s_ = 0.66).	Qualitative and quantitative findings highlight the importance to partnership synergy of distributed decision-making and collaborative approaches to problem-solving.
**Contextual adaptation**	Both partnerships made ongoing adaptations to the interventions to respond to evolving contextual opportunities and threats. The extent of contextual impact and adaptation was far greater in the case of Primary Care Connection (in comparison to Community Health Resources). Primary Care Connection demonstrated synergy in its ability to adapt to its changing context. The capacity to adapt to context was facilitated by having a variety of key stakeholders with medium to high decision-making power in respective organizations, open, timely dialogue about the evolving context and transparent and inclusive processes of decision-making.	The majority of stakeholders in two of the five partnerships (Primary Care Connection (75%) and Service Linkage (67%)) reported that their partnership had been affected *a lot* or *a great deal* by external factors, beyond the control of the partnership. The majority of Primary Care Connection respondents (75%) reported that the partnership had adapted to these external influences *very well* or *extremely well*. Our newly developed sub-scale of External Environment was not correlated with partnership synergy nor any other dimension of partnership work.	Qualitative findings suggest that recognizing and dealing with changes in partnership context are important to achieve partnership synergy. Quantitative findings were limited in terms of assessing the contribution of external context to partnership synergy.

## Data Availability

The raw qualitative data are not publicly available due to privacy restrictions given the small sample and the qualitative nature of inquiry. The raw quantitative data are available at: [63].

## Supplementary Materials

The following are available online at https://www.mdpi.com/article/10.3390/healthcare9081060/s1, Table S1: Mapping of qualitative categories onto the PSAT scales.
