# Supplementary material for: Activating Partnership Assets to Produce Synergy in Primary Health Care: A Mixed Methods Study
Source: Healthcare (Basel). 2021 Aug 18;9(8):1060. doi: 10.3390/healthcare9081060 (PMC8394800; doi:10.3390/healthcare9081060)
Supplement: Supplementary file 1 [file healthcare-09-01060-s001.zip › healthcare-1285420-supplementary.pdf]

Table S1. Mapping of qualitative categories onto the PSAT scales.

Qualitative Categories	Existing PSAT (2002 version) scales	New scales and items	Item content
**1)** Non-financial **(**intangible) assets	**Non-financial resources** (6 items)	New descriptive questions about organizational expertise brought to the partnership table	Assessment of the degree to which the following resources were present: skills and expertise; data and information; connections to target populations; connections to political decision-makers, government agencies, other organizations/groups; legitimacy and credibility; convening power
**2)** Financial **(**tangible) assets	**Financial and other capital resources** (3 items)		Assessment of the degree to which the following resources were present: financial support; space; equipment and goods
**3)** Resource integration	**Efficiency** (3 items), renamed **Resource Utilization**	New descriptive questions about the nature of stakeholder engagement	Assessment of how well the partnership used partner's time, other non-financial resources, and financial resources
**4)** Leadership	**Leadership** (11 items)		Assessment of the partnership's leadership in the following areas:<br>- Taking responsibility for the partnership;<br>- Communicating the vision of the partnership;<br>- Working to develop a common language within the partnership;<br>- Building on the perspectives,<br>resources and skills of partners;<br>- Helping the partnership to look at things differently;<br>- Fostering respect, trust, inclusiveness and openness in the partnership;<br>- Creating an environment where differences of opinion can be voiced;<br>- Inspiring or motivating people involved in the partnership;<br>- Empowering people involved in the partnership;<br>- Resolving conflict among partners;<br>- Recruiting diverse people and organizations into the partnership
**5)** Administration and management	**Administration and management** (9 items)		Assessment of the administration/management's performance in each of the following areas:<br>- Coordinating communication among partners;<br>- Coordinating communication with people and organizations outside the partnership;<br>- Informing partners on how their input is being integrated;<br>- Organizing the overall work of the partnership;<br>- Performing secretarial duties;<br>- Preparing materials that inform partners and help them make timely decisions;<br>- Providing orientation to new partners as they join the partnership;<br>- Minimizing barriers to participation in meetings and activities (e.g., meetings at convenient places and times, providing transportation, etc.);<br>- Facilitating meetings;<br>- Accurately reflecting discussions and decisions in the minutes of meetings or other documentation;<br>- Evaluating the progress and impact of the partnership
**6)** Communication		New scale for **Communication** (3 items)	How the participants learn about what is happening in the partnership; how useful the information is; do the participants receive enough information

**7)** Decision-making	**Decision-making** (3 items)		The top ways in which decisions are made; how comfortable participants are with the way decisions are made; how often participants feel included in the decision-making process; how well the partnership addresses problems.
**8)** Contextual adaptation		New scale for **External environment** (2 items)	Assessment of the extent to which the partnership has been affected by external factors, beyond the control of the partnership; how well the partnership has adapted to these external influences
**9)** Reported benefits	**Benefits of participation** (11 items)<br><br>**Drawbacks of participation** (6 items)		Whether the organization that each stakeholder is representing has benefitted from:<br>- Enhanced ability to address an important issue;<br>- Development of new skills;<br>- Heightened public profile of the organization;<br>- Increased understanding of partners of my organization's expertise and services;<br>- Acquisition of useful knowledge about services, programs or people represented in the partnership;<br>- Enhanced ability to affect public policy;<br>- Development of valuable relationships;<br>- Enhanced ability to meet the needs of my organization's constituency or clients;<br>- Ability to have a greater impact than we could have on our own;<br>- Ability to make a contribution to the community;<br>- Acquisition of additional financial support<br><br>Whether the organization that each stakeholder is representing has experienced the following:<br>- Diversion of time and resources away from other priorities or obligations;<br>- Insufficient influence in partnership activities;<br>- Negative publicity due to association with other partners or the partnership;<br>- Insufficient credit given to my organization for contributing to the accomplishments of the partnership;<br>- Conflict between the demands of this partnership and other projects within my organization
**10)** Partnership synergy	Weiss *et al.* scale [49] – 2 items retained	Jones synergy scale (Jones & Barry, 2011b) – 5 items rateined	How well the partners are able to: identify new and creative ways to solve problems; include the views and priorities of the people affected by the partnership's work. How often the participants feel that the partnership is making good progress towards its goals; added value is achieved as a result of working in this partnership; the skills and unique perspectives of the partners complement each other; all partners are benefiting from the activities of the partnership; how often does the partnership experience feelings of energy, excitement and passion
